# Connaissances et perceptions des enfants d’âge scolaire concernant les géo-helminthiases et la Chimiothérapie Préventive Ciblée dans la Région D'oti au Ghana

**DOI:** 10.48327/mtsi.v2i2.2022.236

**Published:** 2022-05-27

**Authors:** Jean Claude Romaric Pingdwindé OUÉDRAOGO, Joseph W. JATTA, Dennis TABIRI, Mathieu NITIEMA, Mohamed Bonewendé BELEMLILGA

**Affiliations:** 1 School of Public Health, University of Ghana, Legon, Ghana; 2 Département de médecine et pharmacopée traditionnelles, harmacie (MEPHATRA-Ph), Institut de recherche en sciences de la santé (IRSS), Ouagadougou, Burkina Faso; 3 Directorate of Health Research, Ministry of Health, Banjul, The Gambia

**Keywords:** Géohelminthiases, Connaissances, Perceptions, Chimiothérapie préventive, Ghana, Afrique subsaharienne, Soil-transmitted helminthiases, Knowledge, Perceptions, Preventive chemotherapy, Ghana, Sub-Saharan Africa

## Abstract

**Introduction:**

Le programme ghanéen de lutte contre les maladies tropicales négligées visait à sensibiliser la population aux géohelminthiases et à atteindre une couverture de 100 % de la chimiothérapie préventive (CTP) d'ici 2020. Cette étude vise à déterminer les facteurs associés au niveau de connaissances des enfants d’âge scolaire et à décrire leurs perceptions à Krachi East Municipal au Ghana.

**Patients et méthodes:**

Il s'agissait d'une étude transversale à collecte quantitative et dans les ménages, effectuée en juillet-août 2020. Les enfants de 7-14 ans et leurs tuteurs, consentant librement à participer à l’étude, ont été sélectionnés selon un échantillonnage stratifié à deux degrés. Ainsi, 5 communautés rurales et 3 communautés urbaines ont été sélectionnées par échantillonnage aléatoire simple. Puis, un échantillonnage systématique a été appliqué pour sélectionner les enfants et leurs tuteurs dans les ménages. Les statistiques descriptives ont concerné individuellement les variables quantitatives et qualitatives. Une régression logistique binaire uni et multivariée a été réalisée pour déterminer les facteurs associés au niveau de connaissances des enfants, considérant un niveau de signification de 5 %.

**Résultats:**

352 enfants et 352 tuteurs provenant principalement de Dambai (66,48%) ont été interrogés. L’âge médian des enfants était de 11 (IIQ : 9-12) ans et les enfants étaient âgés de 7 à 14 ans. La majorité des enfants étaient de sexe masculin (53,13%), et les tuteurs de sexe féminin (66,48%). Les tuteurs avaient un âge compris entre 15 et 74 ans, avec un âge médian de 36 (IIQ : 30-45) ans. Ils étaient le plus souvent mariés (79,55%). La plupart des enfants ont perçu un bénéfice associé à la CTP (334; 94,89%). La proportion d'enfants percevant un risque associé à la CTP ne différait pas significativement de celle des enfants n'en percevant pas (49,72 % contre 50,28 %; p = 0,8802). Pour les élèves, les enseignants étaient leur principale source d'information sur les vers intestinaux (96,59%). La majorité d'entre eux percevait un soutien de leurs enseignants vis-à-vis de la CTP (96,00%). La proportion d'enfants ne connaissant pas les modes de transmission et les moyens de prévention des vers était respectivement de 41,48 % et de 33,24 %. Globalement, 115 enfants (32,67%) ne connaissaient pas les vers intestinaux. Après cumul des scores de connaissances et classification, les enfants avaient en général une mauvaise connaissance des géohelminthiases et de la CTP (91,19 % contre 8,81 %; p < 0,0001). La bonne connaissance était associée au groupe ethnique [Guan : ORa = 3,96 95%CI 1,11-14,12; p = 0,034], à l’âge de l'enfant [(11-12 ans : ORa = 6,05 95%CI 1,21-30,22; p = 0,026); (13-14 ans : ORa = 8,19 95%CI 1,64-40,89; p = 0,010)] et au sexe des tuteurs (Femme : ORa = 2,97 95%CI 1,02-8,66; p = 0,046) dans le modèle ajusté.

**Conclusion:**

Les plus jeunes enfants et les tuteurs hommes semblent avoir une faible connaissance des vers intestinaux et de la CTP et doivent bénéficier d'une plus grande attention en matière d’éducation sanitaire.

## Abstract (en)

**Background:**

The Ghana Neglected Tropical Diseases control program aimed to raise population awareness on soil-transmitted helminth (STH) infections and achieve a 100% coverage of preventive chemotherapy (PCT) by 2020. This study aims at determining the factors associated with the knowledge of school-age children and describing their perceptions at Krachi East Municipal in Ghana.

**Patients and methods:**

It was a cross-sectional study. Children and their caregivers were selected from 8 communities following a two-stage stratified sampling. Descriptive statistics and binary logistic regression were performed at a 5% significance level.

**Results:**

352 children and their caregivers were surveyed, mainly from Dambai (66.48%). The median age was 11 (IQR: 9-12) years and the children aged 7-14 years. About half of the children were males (53.13%) and most caregivers were females (66.48%). Most children perceived a benefit associated with PCT (94.89%). The proportion of children perceiving a health risk did not differ significantly from those not perceiving a risk (49.72% vs 50.28%; p=0.8802). In general, children had poor knowledge (91.19% vs 8.81%; p<0.0001). Good knowledge was associated with ethnic group [Guan: aOR=3.96 95%CI 1.11-14.12; p=0.034], child age [(11-12 years: aOR=6.05 95%CI 1.21-30.22; p=0.026); (13-14 years: aOR=8.19 95%CI 1.64-40.89; p=0.010)] and caregivers’ sex (Female: aOR=2.97 95%CI 1.02-8.66; p=0.046) in the adjusted model.

**Conclusion:**

Younger children and male caregivers seem to have low knowledge of intestinal worms and PCT. Therefore, they must get more attention regarding health education.

## Background

Worldwide, soil-transmitted helminthiases (STH) affect about 24% of populations living in subtropical and tropical areas, for instance, Sub-Saharan Africa [19]. With a prevalence varying between 1% and 50% depending on the region, they are among the 10 most common diseases in Ghana [5, 7, 8]. STH can cause physical, nutritional, and mental impairment to pre-school and school-age children, among the most at-risk populations [14, 19].

In addition to health education, better access to adequate sanitation and case management, the World Health Organization (WHO) has recommended since 2002 preventive chemotherapy (PCT) to control neglected tropical diseases such as STH in endemic settings [5, 17, 19]. Following this recommendation, the Ghana Neglected Tropical Diseases (NTD) launched its preventive chemotherapy programme against STH in 2007. Up to 2017, Ghana has not reached the 75% target set by the WHO in 2012, like other countries [16, 18]. However, the progress made in implementing the PCT in Ghana has led the NTD programme to target 100% coverage of albendazole once a year for all school-age children, while the WHO targets 75% by 2020 [5, 16]. In effect, preventive chemotherapy (PCT) using albendazole is safe, efficient, and cost-effective in controlling or eliminating STH.

Despite the regular campaigns since 2007 and sustained efforts, STH are still endemic in Ghana, needing at least an annual PCT using an anthelminthic drug [3]. This failure could be due to insufficient coverage or uptake measuring interventions performance or a huge coverage-uptake gap [4, 9]. Lower coverage or uptake could be explained by inadequate communication, knowledge, or perceptions of the children. The perceptions of risk or benefit associated with the PCT and individual knowledge influence drug uptake [10, 15]. It explains why the Ghana Neglected Tropical Diseases Programme also aims to increase children awareness on STH transmission and control [5]. The actual level of school-age children's knowledge on STH and PCT is not known in Ghana. Most studies focused on the prevalence of soil-transmitted helminthiases, and the different species [2, 3]. From the perspective of STH control, it is crucial to know more about children's perceptions and knowledge of worm transmission and prevention.

In Ghana, communities near the Volta Lake – such as Krachi East Municipal – are often endemic for intestinal worms [7]. Therefore, we aimed at describing the perceptions and determining the level of knowledge and associated factors among school-age children in 2019 at Krachi East Municipal in Ghana.

## Patients and Methods

### Study setting

Oti Region is one of the 16 regions in Ghana and is comprised of eight health districts, including Krachi East Municipal. This municipality covers an area of 2,529.4 km^2^ and is bordered to the west by Volta Lake. Its health system is organized into 7 subdistricts (Oti East, Oti West, Katanga-Asukawkaw, Island, Dormabin, Takuroanu, and Kparekpare). The 20 largest communities among these 7 subdistricts, representing at least 54.26% in terms of population, were considered for this study [6]. Krachi East Municipal had 116,804 inhabitants in 2010 and an estimated population of 143,098 in 2019, with a 2.5% average annual growth rate [6]. In 2010, children 5-14 years old were more than 16,894.

**Figure 1:**
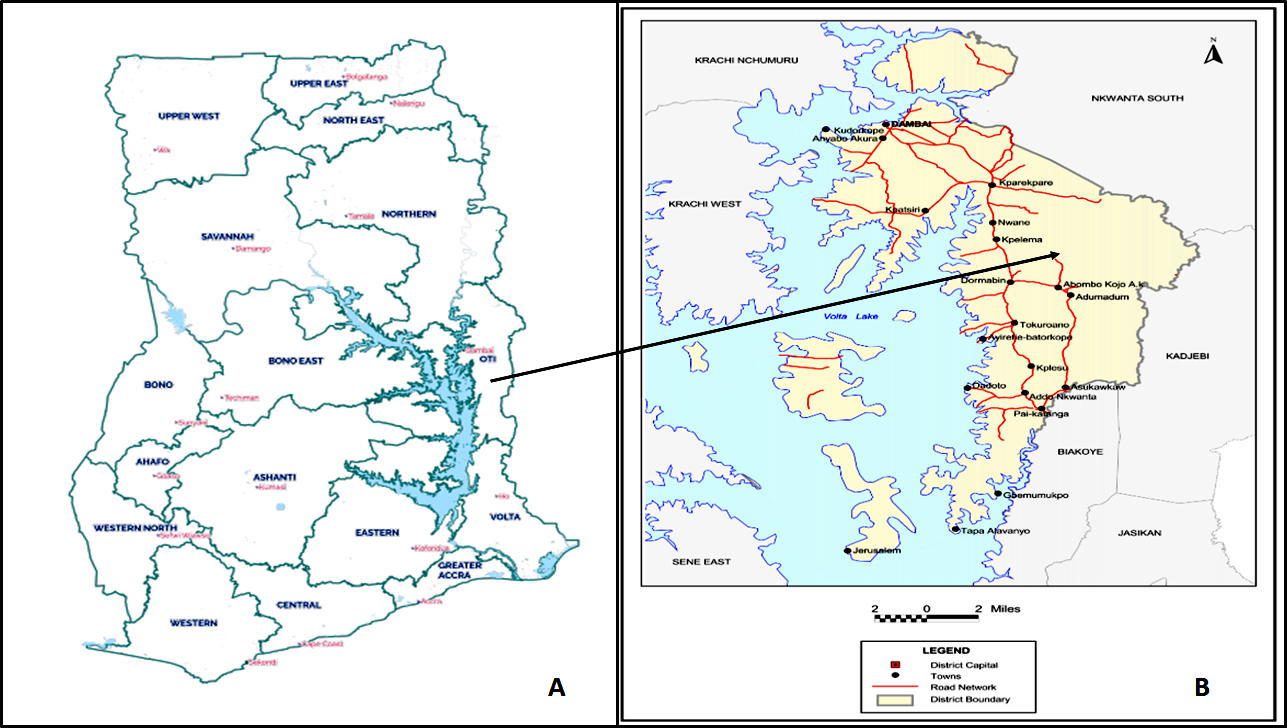
Carte des 16 régions du Ghana (A) et de Krachi East Municipal (B) source : Service de statistiques du Ghana Map of the 16 regions of Ghana (A) and the Krachi East Municipal (B) source: Ghana Statistical Service

The regions of Ghana and the cities of the Municipality are presented at Figure 1.

Soil-transmitted helminthiases and schistosomiasis are endemic in Krachi East Municipal. Integrated preventive chemotherapy (PCT) is done once a year in the endemic areas. Albendazole 400 mg is provided for every qualified child 5-14 years old, while praziquantel dosage is given 40-60 mg/kg body weight statim. Thus, the health and education sectors conducted an integrated PCT, using praziquantel and albendazole, from 4^th^ to 8^th^ November 2019 in the schools. The PCT is school-based, with the teachers being the drug distributors. It was implemented after health education on radio stations, community information centres and letters to institutions to engage the communities.

### Study design

A cross-sectional study was carried out through a community-based data collection in Krachi East Municipal in July-August 2020.

### Study population

The children 7 to 14 years old who lived in Krachi East Municipal in November 2019 were included, along with their caregivers. The children whose caregivers refused the participation to the study were excluded.

### Study variables

Two questionnaires were used in this study: Questionnaire A dedicated to the children, and Questionnaire B addressed to the caregivers and the household's information.

Children's level of knowledge about modes of worm transmission and prevention means was the dependent variable. We first computed the knowledge of transmission and prevention to get a total score of 0-9 based on the awareness of worm transmission (0-3) and preventive measures (0-6). The knowledge of transmission had 3 questions coded 0/1 each: knowledge of the lack of hygiene, human-to-human transmission, and contaminated environment. The knowledge of prevention had 6 questions coded 0/1 each: knowledge of washing hands before eating, washing vegetables/fruits before eating, boiling/preparing raw food before eating, taking drugs, improving human hygiene, and improving environmental hygiene. The computed knowledge was further categorized as Poor knowledge (<3), Good knowledge (4-7 included), High knowledge (>7).

This study considered the following independent variables: sociodemographic variables related to the children and their caregivers; and children's perceptions of STH and PCT.

### Sample size determination

Using the Cochrane formula for estimating a single proportion, a precision level of 0.05, a significance level of 5% for a two-sided test, an anticipated uptake of 70% (5% less than the WHO target of 75% by 2020), the minimum sample size was estimated at 323. With a 10% non-response rate (nr), using this formula: n0/(1-nr) the sample size was set at 359.

### Sampling method

The children were surveyed through stratified community-based sampling. The 20 largest communities reported by the latest Population and Housing survey in 2010 were considered [6]. Based on a threshold of 5,000 inhabitants, the communities were divided into 2 strata: rural and urban. Subsequently, three communities were randomly selected from each stratum by simple ballot: Addo Nkwanta, Dambai and Asukawkaw were urban; Batorkope, Adumadum and Kudorkope were rural. Sikape and Jerusalem communities were randomly sampled from the Island subdistrict – which had 71.5% coverage in schools during the PCT in 2019 – to make 8 communities in total.

In the communities, systematic sampling was conducted. The proportionate size was reported to the total targeted population (children 7-14 years old) of each community to get the sampling interval. From the middle of the housings, the first household with an eligible child was visited. The following households were visited according to the specific sampling interval until the allocated sample size was reached.

### Data collection

Four graduate students were trained on the study proposal and the questionnaire. Then, attempts for consensual translations were made in Twi and Ewe before collecting the data on the field. A pretest was done in Dambai to adjust the questionnaires before the data collection. Questions were mainly translated in Twi, Ewe or kept in English at the participants’ convenience. It was administrated face-to-face using KoBoCollect on a smartphone. The children and their caregivers were surveyed at home, depending on their availability to respond to the questions. The data set was exported as an Excel file for analysis purposes.

### Data analysis

Stata/IC 16 (StataCorp LLC, Tx, USA) software served to analyze the data. Not normally distributed at 5% based on the Shapiro-Wilk test, numeric variables were reported in median (IQR: Q1-Q3). Qualitative variables were summarized into frequency and percentage. The proportions of the risk perceptions and children level of knowledge categories were compared using the maximum likelihood ratio test, at 5% level.

A binary logistic regression was performed, inputting all the variables in the univariate and multivariate analyses, as they were pertinent. The model with the lowest Akaike Information Criteria (AIC) was retained as final. The significance level was set at 5%.

### Ethics approval

The Ghana Health Service Ethical Review Committee approved this study under GHS-ERC 045/02/20 on 25^th^ March 2020. The caregivers were asked for informed assent for his/her child to participate in the study. Assent was also sought from the children 10-14 years old. Illiterate participants had to thumbprint the consent/assent form instead of signing.

## Results

### Coverage and uptake

352 children 7-14 years old were surveyed in Krachi East Municipal. Out of them, 320 received the albendazole during the preventive chemotherapy (PCT) in November 2019 and swallowed it, making an estimated coverage and uptake of 90.91% (95% CI: 87.41%-93.51%) among these school-age children.

### Sociodemographic features

Most children came from the capital city of the Municipality, Dambai (66.48%), and resided in an urban setting (83.81%). Ga-Dagbani (27.56%), Guan (25.28%), and Ewe (22.73%) ethnic groups were the most represented. Among the children, 308 (87.50%) were Christians. Children were almost equally distributed throughout the different age groups, and the median age was 11 (IQR: 9-12) years. Most children were attending primary school (93.75%). Children were mainly males (53.13%), while the caregivers were females (66.48%).

Caregivers’ age varied from 15 to 74 years and the median was 36 (IQR: 30-45) years. They were usually married (79.55%). The sociodemographic figures are shown in Table I.

**Tableau I:** Sociodemographic characteristics of the children and their caregivers Caractéristiques sociodémographiques des enfants et des tuteurs

Characteristics	Frequency	Percentage	Median (IQR: Q1-Q3)
**Community**			
Dambai	234	66.48	
Asukawkaw	40	11.36	
Addo Nkwanta	21	5.97	
Jerusalem	15	4.26	
Sikape	15	4.26	
Kudorkope	10	2.84	
Adumadum	9	2.56	
Batorkope	8	2.27	
**Residence**			
Urban	295	83.81	
Rural	57	16.19	
**Ethnic group**			
Ga-Dagbani	97	27.56	
Guan	89	25.28	
Ewe	80	22.73	
Akan	27	7.67	
Konkomba	23	6.53	
Other	36	10.23	
**Faith**			
Christianity	308	87.50	
Islam	30	8.52	
African religion	14	3.98	
**Child age**			11 (IQR: 9-12) years Range: 7-14 years
7-8	72	20.45	
9-10	95	26.99	
11-12	99	28.13	
13-14	86	24.43	
**Child sex**			
Male	187	53.13	
Female	165	46.88	
**Child level of education**			
None/Kindergarten	2	1.14	
Primary	330	93.75	
Junior high school	18	5.11	
**Caregiver age**			36 (IQR: 30-45) years Range: 15-74 years
**Caregiver sex**			
Male	118	33.52	
Female	234	66.48	
**Caregiver marital status**			
Married	280	79.55	
Not married	72	20.45	
**Caregiver main occupation**			
Agriculture/Forestry	136	38.64	
Services/Sales	93	26.42	
Craft/Related trades	44	12.50	
Fishery	40	11.36	
Student	22	6.25	
Other occupations	17	4.83	

### Children's perceptions of PCT

Schools/teachers were the primary sources of information for the children (96.59%). Children perceiving a health risk associated with the PCT (49.72%) were almost equal to those not perceiving any health risk with the preventive chemotherapy (50.28%); these proportions were not statistically different (p=0.8802). Up to 334 (94.89%) children perceived some health benefit associated with the PCT. More than half of the children perceived that they could get infected with intestinal worms (52.56%). Among the schooling children (n=350), 336 thought they received support from the teachers during the PCT. Those perceptions are presented in Table II.

**Tableau II:** Perceptions des enfants sur la CTP et leurs sources d'information Children's perceptions of PCT and sources of information

	Frequency	Percentage	P-value
**Source of information on STH/PCT**			
School/Teachers	340	96.59	
Radio/TV	7	1.99	
Parents	5	1.42	
**Perceived risk of PCT**			**0.8802**
No	177	50.28	
Yes	175	49.72	
**Perceived benefit of PCT**			
No	18	5.11	
Yes	334	94.89	
**Perceived risk to be infected by intestinal worms**			
No	167	47.44	
Yes	185	52.56	
**Perceived support from teachers (schooling children)**	n=350		
No	14	4.00	
Yes	336	96.00	

### Children's knowledge of intestinal worms and preventive measures

Children's knowledge is presented in Table III. Among the 352 children, 41.48% did not know (score=0/3) about soil-transmitted helminthiases’ transmission, while only 33.24% did not know (score=0/6) the prevention means. The lack of hygiene was the primary transmission risk known by the children (53.69%). To prevent worm infestation getting STH, children reported washing hands before eating (38.07%) and improving human hygiene (24.43%) in general. Overall, 115 children (32.67%) did not know (score=0) about worm transmission and prevention.

**Tableau III:** Connaissances des enfants sur la transmission et les mesures préventives des géohelminthiases Children's knowledge about intestinal worm transmission and preventive measures

Knowledge items	Frequency	Percentage
**Knowledge of worm transmission ways**		
Do not know	146	41.48
Lack of hygiene	189	53.69
Contaminated environment	27	7.67
Human-to-human	20	5.68
**Knowledge of worm prevention means**		
Do not know	117	33.24
Washing hands before eating	134	38.07
Improvement in human hygiene	86	24.43
Taking anthelminthic drugs	51	14.49
Improvement in environment hygiene	22	6.25
Washing vegetables/fruits before eating	20	5.68
Boiling/preparing raw food before eating	15	4.26
Overall lack of knowledge	115	32.67

### Factors associated with the level of knowledge

With a score ≤3, 321 (91.19%) children had poor knowledge versus 31 (8.81%) that showed a good knowledge (score=4-7 included), and 0 with high knowledge (>7). The proportion of children having a poor knowledge was statistically different from those having a good knowledge (p<0.0001). Factors associated with the level of knowledge are shown in Table IV.

**Tableau IV:** Facteurs associés au niveau de connaissances des enfants sur la transmission et la prévention des géohelminthiases Factors associated with children's knowledge level about worm transmission and prevention

Variables	Univariate binary logistic regression		Multivariate binary logistic regression	
	cOR [95% CI]	p-value	aOR [95% CI]	p-value
**Residence**				
Urban	1		1	
Rural	0.33 (0.08-1.44)	0.141	0.28 (0.06-1.35)	0.114
**Ethnic group**				
Ga-Dagbani	1		1	
Guan	2.62 (0.78-8.82)	0.121	1.77 (0.47-6.66)	0.400
Ewe	3.71 (1.13-12.13)	0.030*	3.96 (1.11-14.12)	0.034*
Akan	4.04 (0.94-17.40)	0.061	2.39 (0.48-11.88)	0.287
Konkomba	1.06 (0.11-9.93)	0.961	0.44 (0.04-5.04)	0.512
Other	1.37 (0.24-7.81)	0.725	1.28 (0.17-9.85)	0.815
**Faith**				
Christianity	1		1	
Islam	0.71 (0.16-3.16)	0.657	0.90 (0.14-5.61)	0.907
African religion	0.77 (0.10- 6.10)	0.804	2.46 (0.25-25.02)	0.446
**Child's age**				
7-8	1		1	
9-10	2.36 (0.46-12.05)	0.302	3.74 (0.68-20.63)	0.131
11-12	4.37 (0.94-20.39)	0.060	6.05 (1.21-30.22)	0.026*
13-14	5.68 (1.23-26.27)	0.026*	8.19 (1.64-40.89)	0.010*
**Child's sex**				
Male	1		1	
Female	0.80 (0.38-1.69)	0.564	0.49 (0.21-1.15)	0.102
**Caregiver's age**	1.00 (0.97-1.03)	0.973	1.00 (0.97-1.03)	0.886
**Caregiver's sex**				
Male	1		1	
Female	2.23 (0.89-5.60)	0.087	2.97 (1.02-8.66)	0.046*
**Caregiver's marital status**				
Not married	1		1	
Married	1.68 (0.74-3.82)	0.219	0.65 (0.26-1.63)	0.357
**Perceived risk of MDA**				
No	1		1	
Yes	0.61 (0.29-1.30)	0.203	0.65 (0.28-1.50)	0.310
**Perceived risk of infection**				
No	1		1	
Yes	0.63 (0.30-1.32)	0.218	0.51 (0.23-1.14)	0.102

N: 352; p= 0.0436; Pseudo-R2: 0.1340; AIC=217.71

From the univariate analysis, ethnic group and child age were significantly associated with children's knowledge. Ewe people showed a 3.71 times higher chance of good knowledge than the Ga-Dagbani group (p-value=0.030). Children aged 13-14 years had 5.68 times higher odds of good knowledge than those 7-8 years (p-value=0.010). In contrast, Akan people had about 4 times higher chance of good knowledge than Ga-Dagbani people; this was not statistically significant (p=0.061).

While adjusting for confounders, caregivers’ sex, ethnic group and child age were associated with children's knowledge. Thus, Ewe people still have increased odds of knowledge than the Ga-Dagbani (p-value=0.034) and the children 13-14 years compared to those 7-8 years. In addition, the children 11-12 years old had about 6 times increased likelihood of knowledge than those 7-8 years. Also, children whose caregivers were females showed a 3 times higher chance of knowing about worms/PCT than those whose caregivers were males (p-value=0.046).

## Discussion

This study aimed at describing children's perceptions about soil-transmitted helminthiasis and determining the level of knowledge and the associated factors among the children 7-14 years in Krachi East Municipal in Ghana. Almost half of the children perceived a risk associated with mass drug administration (MDA) while the other half perceived no risk. In contrast, 94.89% of the children perceived a benefit associated with MDA. A few numbers of children (8.81%) showed a good knowledge of worm transmission and prevention, and this was in fact associated with children's age and ethnic group, and caregivers’ sex.

The uptake of anthelminthic administration is likely to be high, as most children in this study believed that preventive chemotherapy is beneficial to their health. Perceptions of potential benefit of MDA campaigns were also known to favor drug uptake against lymphatic filariasis [10]. Conversely, the perceptions of a risk associated with the MDA would endanger individuals’ compliance with anthelminthic drug [15]. Although most children did not perceive a risk, an important proportion of them believed in health risk associated with swallowing the anthelminthic drug. These negative perceptions could prevent the children from getting and swallowing such drugs. Fortunately, schooling children trusted their teachers’ support. It is then good that the PCT against STH was school-based, with the teachers being the distributors. Support from teachers positively influences the uptake of PCT drugs [12]. However, the negative perceptions need to be addressed by intensifying health education through radio stations, community information centers and letters to institutions to engage the communities.

As for the uptake of interventions targeting STH, studies evaluating children's knowledge on STH symptoms, transmission ways, and prevention strategies are scarce. Most people focused on the other Neglected Tropical Diseases (NTD) like lymphatic filariasis, schistosomiasis and malaria. This study on STH found that children knew more about STH prevention than the transmission ways (66.76% vs 58.52%). This trend was also reported for STH in Zimbabwe, contrary to schistosomiasis, for which they knew more about the causes (32% vs 22.1%) [11]. In Nigeria, most school-age children showed awareness of STH contamination ways [1]. In this study, fewer children knew about STH prevention and transmission than reported among children (75.6% vs 81.9%, respectively) in India [13]. Children's knowledge was very poor in this study, as only 31 children out of 352 had good knowledge (4-7 points included). That is a catastrophic situation which commands that the Ghana Neglected Tropical Diseases Programme takes specific actions to raise children's awareness. In fact, knowledge is key at the individual level in one decision to comply with anthelminthic treatment [15]. Knowledge on soil-transmitted helminthiases’ prevention and transmission is likely to influence children's perceptions, hopefully towards a better uptake. It is then crucial to look at the potential facilitators and barriers to this knowledge.

In this study, the likelihood of good knowledge increased with children's age, and the difference with the 7-8 year-olds was significant for the 11-12 and 13-14 year-olds [(11-12 years: aOR=6.05 95%CI 1.21-30.22; p=0.026); (13-14 years: aOR=8.19 95%CI 1.64-40.89; p=0.010)]. Most participants were attending school (99.43%) and have already studied intestinal worms, their contamination routes, and preventive measures. It is not surprising they knew more than the younger children. As a result, the Ghana NTD programme should consider specific actions during the PCT to raise younger children's awareness of STH transmission and/or prevention. In addition, children whose caregivers were females were likely to have good knowledge. It is so because females frequently go to the hospital for them and their infants, benefitting from education from health workers. If females’ knowledge has to be reinforced, males should be targeted for specific health education concerning STH, its contamination routes and preventive measures.

Given the importance of negative beliefs regarding the preventive chemotherapy benefits, as well as children's low knowledge, important recommendations can be formulated. As the intervention is school-based, health staff should train the teachers to improve their understanding of the preventive chemotherapy. The health and education staffs should collaborate in educating the children – especially the youngest – on STH prevention and transmission through dedicated activities. The staffs must target the communities to engage male caregivers in issues related to the preventive chemotherapy against soil-transmitted helminthiases. With the Ghana Neglected Tropical Diseases Programme, the health and education staff should improve the education through radio stations and community information centres to better engage non-schooling children.

To the best of our knowledge, this study is the first to evaluate the perceptions and knowledge of school-age children regarding STH prevention and transmission in Ghana. Raising children's awareness of STH was a key objective in the latest strategic plan of the Ghana NTD programme. The perceptions and the level of knowledge were estimated in this study, results that will allow adjusting the intervention based on the factors determined. A key limitation is that the study was done a year after the intervention was done (2019); then the knowledge and perceptions may have changed with time. The present study was a cross-sectional study, such that inferences on causality cannot be made. Although the biggest city (Dambai) was selected, the 20 largest communities represented 54.26% of the municipality's population from which the sample was done. This may not be representative enough of the overall situation. Also a complete evaluation of caregivers’ socioeconomic status was not done, while it would have improved the interpretation of the results. Finally, the study enrolled only two non-schooling children; hence the factors associated with STH/PCT knowledge may not be valid for those children.

## Conclusion

Since 2008 at last, the Ghana Neglected Tropical Diseases Programme has implemented preventive chemotherapy to control soil-transmitted helminthiases once a year. The knowledge of school-age children on STH and the PCT intervention is key to getting 100% coverage. Unfortunately, their knowledge is poor in general. The programme should intensify health education targeting male caregivers and younger children during the PCT and at any time to raise their awareness on the subject. This study gives baseline information on school-age children's knowledge for the Ghana NTD programme to take further actions.

## List of Abbreviations

NTD: Neglected tropical diseases PCT: Preventive chemotherapy STH: Soil-transmitted helminthiasis MDA: Mass drug administration

## Funding

The initial study was funded by the Special Programme for Research and Training on Tropical Diseases (TDR/WHO). The funder had no role in the design, conduct, data analysis of this study, and decision to submit the manuscript.

## Competing Interests

The authors declare that they have no competing interests.

## Authors Contribution

JCRPO designed the study, acquired, analyzed the data, and drafted the manuscript. JWJ and DT contributed substantially to interpreting the data, drafting, and critically reviewing the manuscript. MN and MBB contributed to the review of the draft manuscripts. All authors read and approved the final manuscript.

## Appendix - Questionnaire A: Questions addressed to children

**Table:**

Q1	Ask for the years at the last birthday	/__/__/ (Years)
Q2	Ask for the sex	Male /__/ Female /__/
Q3	Are you going to school?	Yes /__/ No /__/
Q3a	At which level of education are you? (For schooling only)	Primary /__/ Junior high school /__/ Other /__/
Q3b	Which school are you attending? (For schooling only)	
Q3c	Do you have a functional toilet at the school? (For schooling only)	Yes /__/ No /__/
Q4	Where do you usually go when you want to defecate? (For all)	Toilet /____/ Open defecation /__/ Other /__/
Q5a	According to you, how worms are transmitted? (Read the question and wait for answers)	Don't know /__/ Contaminated food and water/__/ Dirty hands /__/ Lack of hygiene /__/ Other /___/
Q5b	In your opinion, how to avoid having worms? (Read the question and wait for answers)	Don't know /__/ Wash hands before eating /__/ Wash vegetables before eating /__/ Taking drugs /__/ Improvement of hygiene /__/ Other /___/
Q6	Which one(s) is (are) your primary source(s) of information on worms and or MDA?	School/Teachers /__/ Parents /__/ Radio-TV /__/ Other /___/
Q7	Do you think that there is a risk associated with participation in a mass drug administration?	Yes /__/ No /__/ Don't know /__/
Q8	Do you think that there is a benefit associated with participation in a mass drug administration?	Yes /__/ No /__/ Don't know /__/
Q9	Do you think that worms can infect you?	Yes /__/ No /__/ Don't know /__/
Q10	Did you receive any drug during the last MDA campaign in October 2019?	Yes /__/ No /__/ Don't know /__/
Q10a	In your opinion, why didn't you receive a drug? (Only for those you answered No/Don't know at the previous question)	
Q11	Did you swallow the drug you received? (Only for those who received the drug)	Yes /__/ No /__/ Don't know /__/
Q11a	Why didn't you swallow the drug you received? (Only for those who answered No/Don't know at the previous question)	
Q12	Do you think your teacher supports you in taking the drug during MDA? (Only for current students)	Yes /__/ No /__/ Don't know /__/

## Appendix - Questionnaire B: Questions addressed to the caregivers

**Table:**

Q13	What is your age, please?	/__/__/__/
Q14	Sex of the parent or caregiver been interviewed	Male /__/ Female /__/
Q15	What is your current marital status, please?	Single /__/ Married/Cohabiting /__/ Divorced/Widowed /__/ Other /___/
Q16	What is your main occupation?	Farming /__/ Trading /___/ Housewife /__/ Unemployed /__/ Other /__/
Q17	What is your highest level of formal education?	None /__/ Primary /__/ Junior/senior secondary /__/ Technical/Vocational /__/ Tertiary /__/
Q18	What is your religious affiliation?	Christianity /__/ Islam /__/ Traditionalism /__/ Other /__/
Q19	Living environment	Urban /__/ Rural /__/
Q20	Community of residence	
Q21	What is the approximate distance between your house and the Volta river?	/__/__/__/ (km)
Q22	Do you have a functional toilet at home?	Yes /__/ No /__/
Q22a	According to you, how worms are transmitted? (Read the question and wait for answers)	Don't know /__/ Contaminated food and water/__/ Dirty hands /__/ Lack of hygiene /__/ Other /__/
Q22b	In your opinion, how to avoid having worms? (Read the question and wait for answers)	Don't know /__/ Wash hands before eating /__/ Wash vegetables before eating /__/ Taking drugs /__/ Improvement of hygiene /__/ Other /__/
Q24	According to you, is there any risk or benefit in participating in MDA?	No /__/ Risk /__/ Benefit/__/ Both /__/ Don't know /__/
Q25	What is your attitude towards MDA?	Indifferent /__/ Positive /__/ Negative /__/
Q26	In your view, why your child did not receive the drug during the 2019 MDA? (Only for children who answered No/Don't know at Q10)	………………………… ………………………… …………………………
Q27	According to you, why your child, who received the drug during the 2019 MDA, did not swallow it? (Only for children who answered No/Don't know at Q11)	………………………… ………………………… …………………………
Q28	After swallowing the drug, did your child have any adverse/side effects? (Only for children who responded Yes at Q11)	Yes /__/ No /__/ Don't know /__/ If yes …………………………
